# Deep Learning for Smartphone-Based Malaria Parasite Detection in Thick Blood Smears

**DOI:** 10.1109/JBHI.2019.2939121

**Published:** 2019-09-23

**Authors:** Feng Yang, Mahdieh Poostchi, Hang Yu, Zhou Zhou, Kamolrat Silamut, Jian Yu, Richard J. Maude, Stefan Jaeger, Sameer Antani

**Affiliations:** Beijing Key Lab of Traffic Data Analysis and Mining in School of Computer and Information Technology, Beijing Jiaotong University, Beijing 100044, China, and also with the Lister Hill National Center for Biomedical Communications, National Library of Medicine, NIH, Bethesda, MD 20894 USA; Lister Hill National Center for Biomedical Communications, National Library of Medicine, National Institute of Health, Bethesda, MD 20894 USA; Lister Hill National Center for Biomedical Communications, National Library of Medicine, National Institute of Health, Bethesda, MD 20894 USA; Beijing Key Lab of Traffic Data Analysis and Mining in the School of Computer and Information Technology, Beijing Jiaotong University, Beijing 100044, China; Mahidol-Oxford Tropical Medicine Research Unit, Bangkok 10400, Thailand; Beijing Key Lab of Traffic Data Analysis and Mining in the School of Computer and Information Technology, Beijing Jiaotong University, Beijing 100044, China; Mahidol-Oxford Tropical Medicine Research Unit, Bangkok 10400, Thailand; Lister Hill National Center for Biomedical Communications, National Library of Medicine, National Institute of Health, Bethesda, MD 20894 USA; Lister Hill National Center for Biomedical Communications, National Library of Medicine, National Institute of Health, Bethesda, MD 20894 USA

**Keywords:** Deep learning, convolutional neural networks, computer-aided diagnosis, malaria

## Abstract

**Objective:**

This work investigates the possibility of automated malaria parasite detection in thick blood smears with smartphones.

**Methods:**

We have developed the first deep learning method that can detect malaria parasites in thick blood smear images and can run on smartphones. Our method consists of two processing steps. First, we apply an intensity-based Iterative Global Minimum Screening (IGMS), which performs a fast screening of a thick smear image to find parasite candidates. Then, a customized Convolutional Neural Network (CNN) classifies each candidate as either parasite or background. Together with this paper, we make a dataset of 1819 thick smear images from 150 patients publicly available to the research community. We used this dataset to train and test our deep learning method, as described in this paper.

**Results:**

A patient-level five-fold cross-evaluation demonstrates the effectiveness of the customized CNN model in discriminating between positive (parasitic) and negative image patches in terms of the following performance indicators: accuracy (93.46% ± 0.32%), AUC (98.39% ± 0.18%), sensitivity (92.59% ± 1.27%), specificity (94.33% ± 1.25%), precision (94.25% ± 1.13%), and negative predictive value (92.74% ± 1.09%). High correlation coefficients (>0.98) between automatically detected parasites and ground truth, on both image level and patient level, demonstrate the practicality of our method.

**Conclusion:**

Promising results are obtained for parasite detection in thick blood smears for a smartphone application using deep learning methods.

**Significance:**

Automated parasite detection running on smartphones is a promising alternative to manual parasite counting for malaria diagnosis, especially in areas lacking experienced parasitologists.

## I. Introduction

MALARIA is a worldwide life-threatening disease. According to the 2018 World Health Organization (WHO) malaria report [1], about 219 million malaria cases were detected worldwide in 2017, causing approximately 435,000 deaths. Microscopy examination of stained thick and thin blood smears is the gold standard for malaria diagnosis [2], [3]. Microscopy examination is low-cost and is widely available, but is time-consuming. Moreover, the effectiveness of microscopy diagnosis depends on the parasitologists’ expertise [4]. It is very common for parasitologists to work in resource-limited environments, with no rigorous system in place that can ensure the maintenance of their skills or/and diagnostic quality. This leads to incorrect diagnostic results and thus inappropriate treatment [4]. For example, false positive diagnostic results lead to unnecessary use of anti-malaria drugs and suffering from their side effects such as abdominal pain, nausea, etc., whereas false negative diagnosis leads to unnecessary use of antibiotics, second consultation, and potential progression of more severe malaria [5]. Therefore, the development of an automated system for malaria diagnosis is an appealing research goal for improving individualized patient treatment and management. Automated parasite detection has two big advantages: 1) it can provide a more reliable diagnosis, especially in resource-limited areas, and 2) it reduces diagnostic costs. Parasite counts are essential to diagnosing malaria and quantifying disease severity. They are also important for monitoring patients to measure drug-effectiveness and potential drug-resistance. In this study, we investigate automatic malaria parasite detection and counting in digital images of thick blood smears acquired with smartphones.

**Fig. 1.**
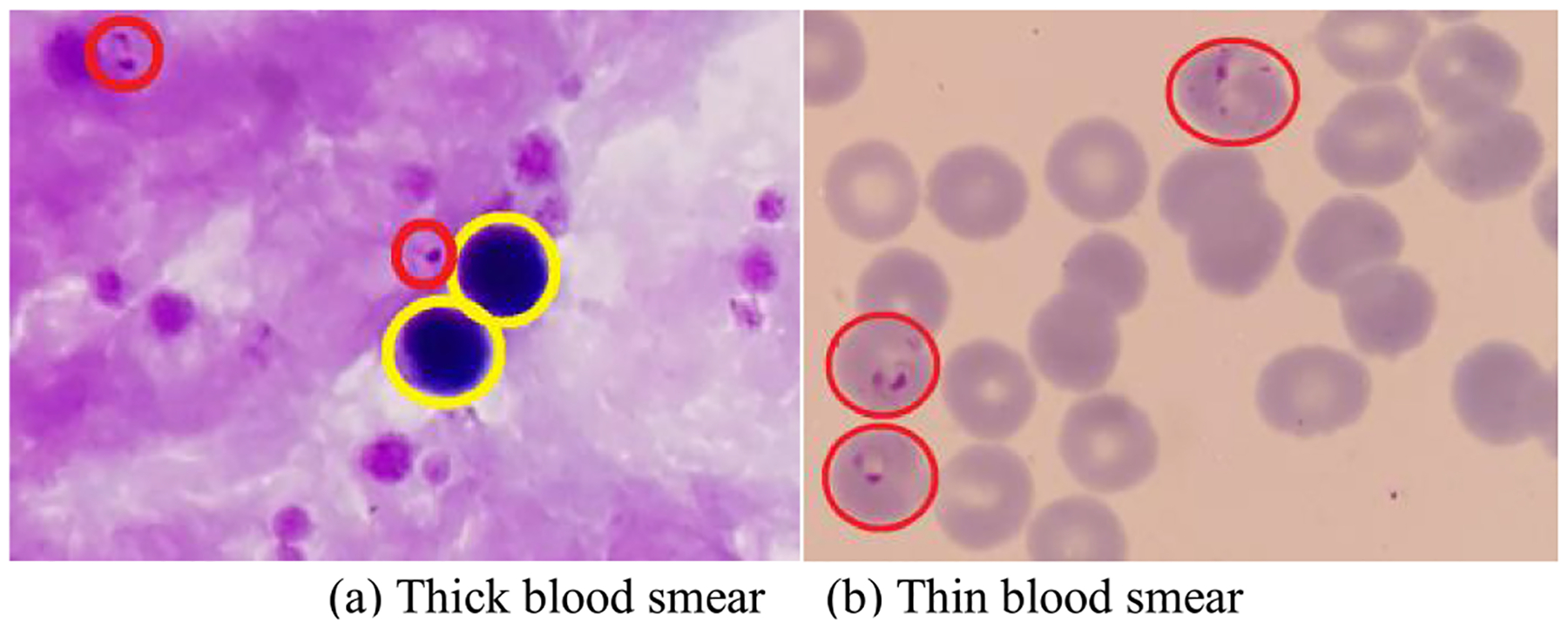
Examples of thick and thin blood smears. Red circles are parasites and yellow circles are white blood cells.

A thick blood smear is used to detect the presence of malaria parasites in a drop of blood. It allows more efficient detection of parasites than a thin blood smear, with about 11 times higher sensitivity [5]. A thin blood smear results from spreading a drop of blood across a glass slide, and is typically used to differentiate parasite species and development stages. Thick and thin blood smears, as shown in Fig. 1, require different processing methods for parasite detection. In thin blood smears, both white blood cells (WBCs) and red blood cells (RBCs) are clearly visible. A typical step for automatic parasite detection in thin smears is to first segment RBCs and then classify each segmented RBC as infected or uninfected [5]–[7]. In thick blood smears, however, only WBCs and the nuclei of RBCs are visible (see Fig. 1(a)). Therefore, parasites need to be detected directly, and a typical step is to first preselect parasite candidates and then classify the candidates as either actual parasites or background noise. This can be challenging because the nuclei of WBCs and various non-parasite components can absorb stain, creating artifacts that can lead to false parasite detection.

### A. Related Work

In recent years, several approaches have been proposed for image processing and analysis on both thin and thick blood smears, aiming at automated detection of parasites. Reviews of the published literature may be found in [5], [8], [9]. In the following paragraph, we provide a brief overview of the approaches for malaria detection in thick blood smears.

Traditional parasite detection techniques are often performed based on segmentation [10]–[13] using thresholding and morphological operations. Kaewkamnerd *et al*. [10] propose a method using an adaptive threshold on the V-value histogram of the HSV image to extract parasite candidates and white blood cells (WBCs) from the background, and then distinguish parasites from WBCs according to their size. Evaluation on 20 images shows that the proposed method achieves an accuracy of 60%. Hanif *et al*. [11] use an intensity-stretching method to enhance the contrast of 255 thick blood smears, and then use an empirical threshold to segment malaria parasites. The authors show qualitative results on different images, in which different empirical thresholds are applied to obtain satisfying segmentation results. Chakrabortya *et al*. [12] combine a morphological segmentation with color information to identify parasites in thick blood smears. Experiments are performed on 75 images and patch level evaluation shows a successful detection rate of 95% with a false positive ratio of 10%. Dave *et al*. [13] perform histogram-based adaptive thresholding and morphological operations on denoised images to detect RBCs infected by malaria parasites in thin and thick blood smears. Patch level evaluation on 87 images shows that the method detects 533 parasites compared to 484 parasites annotated as ground truth. Traditional approaches for parasite detection are simple and fast, whereas they are difficult to extend to large datasets. This is due to the fact that traditional approaches are very sensitive to image variations and that parameters are very often determined empirically. Performance evaluation on patch level on small datasets (from 20 to 300 images) can change greatly when evaluating on big datasets, on image level or patient level.

Feature-based approaches involve feature extraction and classification based on machine learning techniques [14]–[18]. Elter *et al*. [14] extract 174 features from pre-detected plasmodia candidates and apply a Support Vector Machine (SVM) classifier to the feature set for parasite identification. The authors report a sensitivity of 97% for 256 images on patch level. Purnama *et al*. [15] extract features from histograms of RGB channel, H channel from HSV space, and H channel from HIS space, and then use Genetic Programming to identify parasite type and stage. Their classification model on 180 patches achieves an average accuracy of 95.58% for parasite identification and 95.49% for non-parasite identification. Yunda *et al*. [16] extract color features, co-occurrence texture features, and wavelet-based texture features from the pre-segmented image, and then use Principal Component Analysis (PCA) to reduce redundant features, followed by a neural network model for the final classification. Evaluation on 110 images shows that the sensitivity for parasite detection is 76.45%. Quinn *et al*. [17] propose to first split each image into 475 randomly overlapping patches using downsampling and sliding window screening, then extract connected component and moment features from the patches, and finally use a randomized tree classifier for the classification. The method is evaluated on 2903 images from 133 patients and produces a precision of 90% at a recall of 20% on patch level. Rosado *et al*. [18] use an adaptive thresholding approach for the parasite detection and then apply geometry, color and texture features in combination with a RBF kernel based SVM classifier for WBC and parasite identification. Evaluation on 94 images from 6 patients shows their automatic prediction of parasites has achieved a patch level accuracy of 91.8% along with a sensitivity of 80.5% and a specificity of 93.5%, while their WBC detection achieves 98.2% sensitivity and 72.1% specificity. The feature-based approaches evaluate their performance on patch level. That is, the input sample is a single patch image and the evaluation is typically a patch classification accuracy. However, the ultimate goal for malaria patient diagnosis is to detect and classify all patches (both parasites and false positives) for a patient. A satisfying patch level classification performance does not necessarily assure good performance on image level or patient level.

Deep learning is the latest trend in machine learning for its superior performance on big data. It has already boosted the performance in many non-medical areas. Recently, deep learning has gained increasing recognition in computer-aided diagnostic systems. Two main factors contributed to this development: 1) compared to traditional methods and feature-based approaches, deep learning requires neither segmentation nor handcrafted features, offering an end-to-end solution and 2) it can discover hierarchical feature representations that are solely derived from data [19]–[21]. In the past three years, deep learning methods have been applied to thick blood smears for automatic feature extraction and parasite detection. Delahunt *et al*. [22] propose a combination of a linear SVM and a Convolutional Neural Network (CNN) for classification after localizing parasite candidates. They suggest the use of CNNs for feature extraction, whereas their reported results on 143 patients use only traditional features (including morphological, color, texture and Harr-like features), showing the method predicted a Limit of Detection (LoD) about 300 parasites/*μ*L at a specificity of 92% on patient level. Quinn *et al*. [23] propose a parasite detection by training a four-layer CNN model on overlapping patches, which are extracted from the downsampled RGB images. They report an average precision of 97% on 1182 images. However, the authors split the training dataset and testing dataset on image level and not on patient level. That is, their training dataset and testing dataset may include images from the same patient. The performance is evaluated only on patch level. Mehanian *et al*. [24] first detect WBCs by applying a Gaussian-kernel SVM on thresholded candidates, then train another Gaussian-kernel SVM on the parasite candidates, which are generated using a dynamic local thresholding method, and finally use a CNN model for feature extraction and classification. The authors report that their method achieves a sensitivity of 91.6% and a precision of 89.7% at a specificity of 94.1% on patch level, and a LoD of about 100 parasites/*μ*L at a specificity of 90.0% on patient level based on 1452 images from 195 patients. However, a run time within roughly 20 minutes (on a quad-core processor) for parasite detection is not necessarily faster than human processing. Torres *et al*. [25] test Autoscope (a prototype digital microscope device with automated methods proposed in [24]) at two peripheral health facilities, with routine microscopy and Polymerase Chain Reaction (PCR) as reference standards. The authors conclude that Autoscope’s performance (sensitivity of 72% at a specificity of 85%) is close to that of routine microcopy (sensitivity of 68% at a specificity of 100%) when the slides had adequate blood volume; otherwise, its performance will be less than routine microscopy.

Table I is an overview of the existing parasite detection approaches that have been applied to thick blood smears. The traditional parasite detection techniques in [10]–[13] use small datasets and do not achieve high accuracies. Experimental evaluations of the methods are either qualitative or on patch level, which does not guarantee similar results on patient level. Feature-based approaches [14]–[18] generally achieve a better detection rate of parasites than the traditional techniques. However, most of them [14]–[16], [18] use datasets with less than 256 images, and all evaluations are on patch level. An evaluation of such approaches on patient level may result in a big performance drop. A big dataset has been used in [17]; however, the authors only achieve a sensitivity of 20%. So far, only four papers [22]–[25] have used deep learning methods for parasite detection on big datasets in thick blood smears, and three of them [22], [24], [25] have performed evaluation on patient level. In the literature mentioned above, only three papers [17], [18], [23] have worked on smartphone-acquired images for thick smears. Among these papers, [17], [23] use the smartphone only for data acquisition with the intention to process these images on a more powerful platform for remote diagnosis. Reference [18] is the only work that has implemented a screening application on a smartphone. The goal of our paper is to develop a parasite detection application for smartphones based on deep learning, which can provide a better performance compared to the traditional SVM, as implemented in [18].

### B. Contributions

Compared to the existing work for thick blood smear processing, we make the following contributions: First, we develop a smartphone system for automated parasite detection in thick blood smears based on our proposed intensity-based Iterative Global Minimum Screening (IGMS) method for fast automatic preselection of parasite candidates and our customized CNN model [19]–[21], [26]–[28] for classification of parasite candidates as either parasites or background. To the best of our knowledge, this is the first work designed for parasite detection in smartphones for thick blood smears based on deep learning methods. Second, our system is fast. It takes about 10 seconds to detect parasites in a 3024 × 4032 image on a regular Android smartphone. Third, we test our approach on a much larger image set acquired from 150 patients, including 1819 thick smear images and 84,961 annotated parasites, which we release publicly together with this paper.

We organize the rest of the paper as follows: Section II presents the details of our proposed method for automated parasite detection. Section III introduces the dataset, the experimental setup, and results. In Section IV, we discuss our results followed by a conclusion in Section V.

## II. Methods

**Fig. 2.**
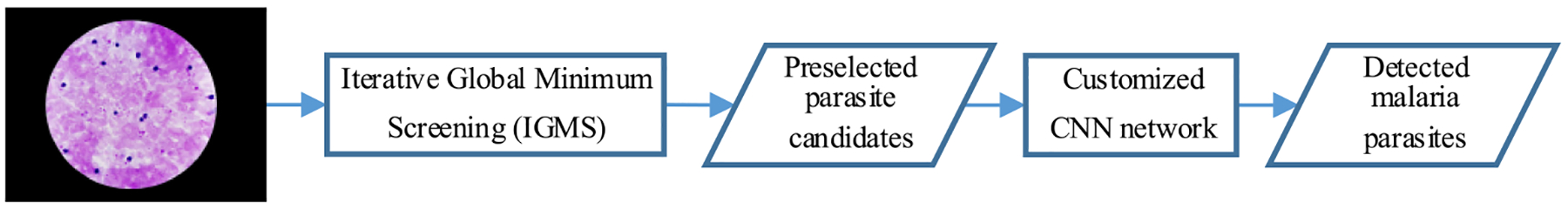
Pipeline of the proposed system for automated parasite detection.

Splitting our problem into a screening and classification step allows faster processing because we only need to predict on a relatively small number of pixel patches, which reduces the overall processing cost. We illustrate the pipeline of our method in Fig. 2.

### A. Parasite Candidate Screening

**Fig. 3.**
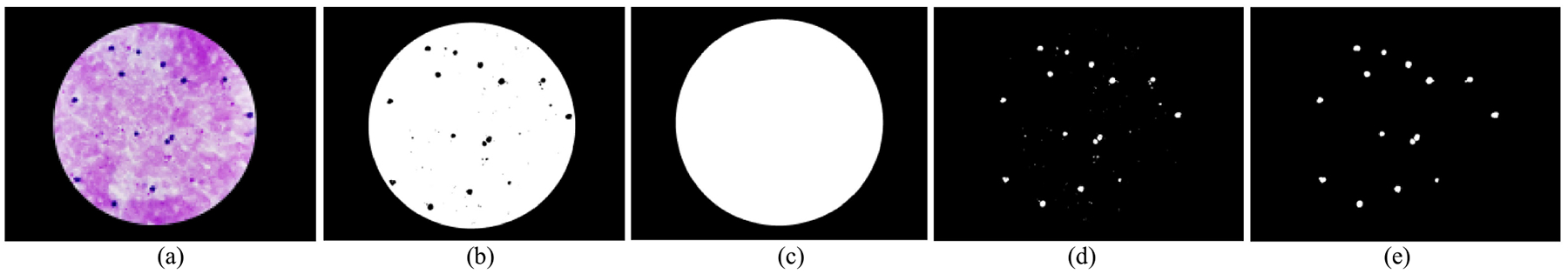
Example of WBC detection. (a) A sample slide image of a thick blood smear acquired with a smartphone. (b) Detected objects after using Otsu thresholding. (c) Detected field of view ROI mask. (d) Detected WBCs including small areas of noise. (e) Detected WBCs after filtering noise in (d).

The screening stage reduces the size of the initial search space and preselects a subset of most likely parasite candidates. Parasite candidates are selected according to the lowest intensities in grayscale based on a histogram analysis, exploiting that the nuclei of parasites and WBCs have darker intensities than the background (Fig. 3(a)). To eliminate WBC distraction, we filter out WBCs before performing the parasite candidate screening. Therefore, our intensity-based screening method for parasite candidate preselection consists of WBC detection and parasite candidate generation. The WBC detection first filters all WBCs present in the image. Then, the parasite candidate generation produces regions of interest by localizing the lowest intensities across a thick blood smear image.

#### 1) WBC Detection:

A sample smear image is shown in Fig. 3(a). We first convert the RGB image into a grayscale image. Then, we convert the grayscale image into a binary mask *M*_1_ using Otsu’s method [29]. In this binary mask *M*_1_, the large ROI area corresponding to the field of view is shown as foreground (white) while WBCs are shown as background (dark); see Fig. 3(b). By filling the holes inside the large field of view ROI area, we obtain the field of view mask *M*_2_, shown in Fig. 3(c). WBCs can then be separated out by subtracting the binary mask *M*_1_ from the ROI mask *M*_2_ (see Fig. 3(d)). Clean WBCs are finally obtained by filtering small noisy areas. Fig. 3(e) demonstrates the result of this step. The pixels of WBCs are set to zeros for the following parasite detection step.

#### 2) Parasite Preselection Using Iterative Global Minimum Screening (IGMS):

**Fig. 4.**
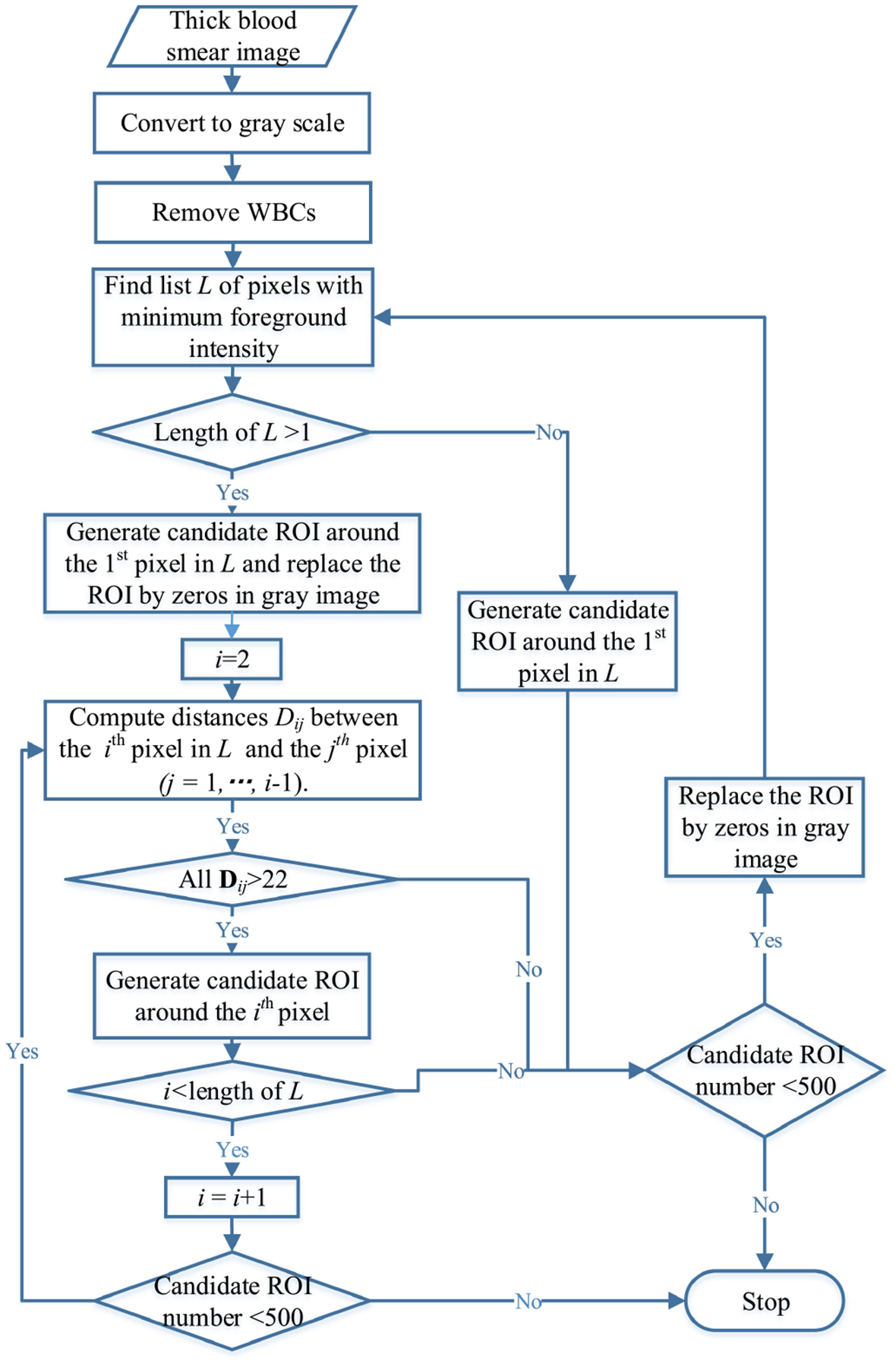
Flowchart of parasite candidate screening (IGMS method).

**Fig. 5.**
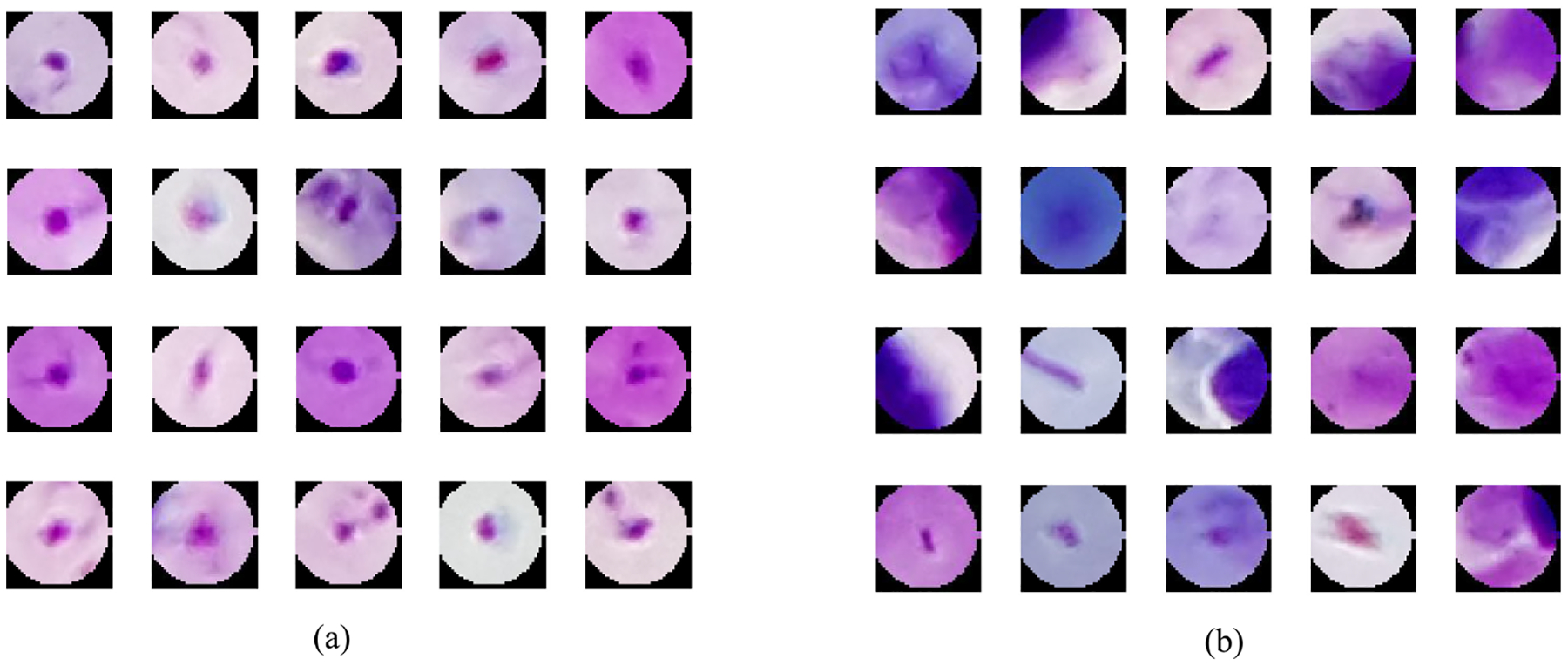
Parasite candidates generated by the IGMS method. (a) 20 positive patches. (b) 20 negative patches.

IGMS generates RGB parasite candidates by localizing the minimum intensity values in a grayscale image. If only one pixel is localized, a circular region centered at this pixel location with a pre-defined radius of 22 pixels (average parasite radius) is cropped from the original RGB image and is selected as a parasite candidate (Fig. 5(a)). If more than one pixel is localized, a new parasite candidate centered at the *i*th pixel is added when all the distances between the *i*th pixel and previously selected pixels are larger than 22. Once a parasite candidate is selected, the intensity values inside this region of the grayscale image will be replace by zeros to guarantee the convergence of the IGMS method. The screening stage stops when the number of parasite candidates reaches a given number. In our experiments, we select 500 parasite candidates for each image to cover the true parasites as much as possible. Experiments on our dataset of 150 patients show that we can achieve a sensitivity above 97% on patch level, image level, and patient level when using this number. Each parasite candidate is a 44 × 44 × 3 RGB patch image, with pixels having a distance greater than 22 to the center set to zero. Fig. 4 shows the processing flowchart for IGMS and Fig. 5 shows examples of positive and negative patches extracted by IGMS.

### B. Parasite Classification

**Fig. 6.**
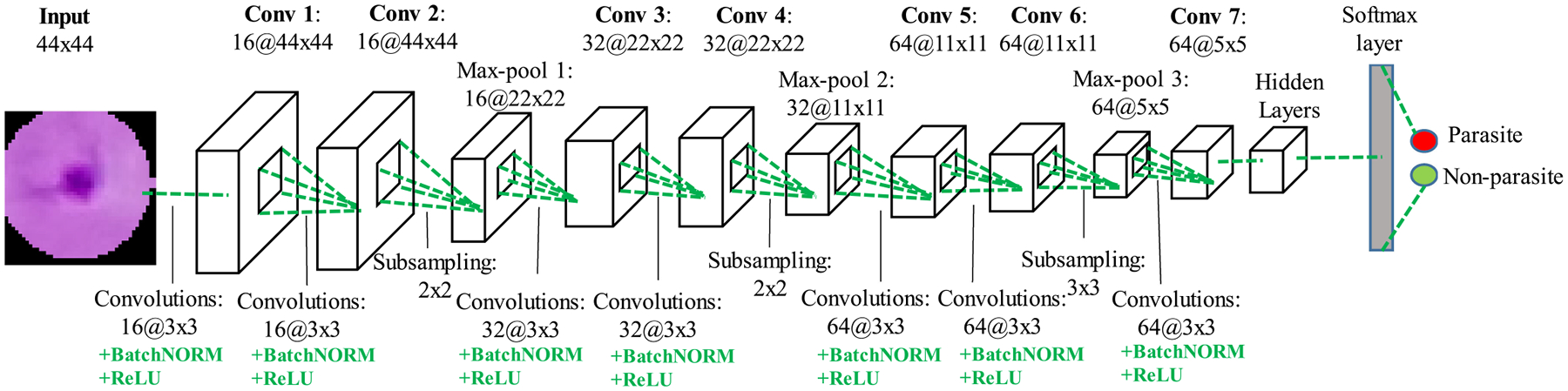
Architecture of the customized CNN model for parasite classification. Conv, Max-pool and BatchNorm denote convolution, max-pooling and batch normalization, respectively. The numbers above the cuboids indicate the dimensions of the feature maps. The numbers below the green dotted line represent the convolutional kernel sizes and the sizes of the max-pooling regions. The hidden layers include three fully connected layers and two dropout layers with a dropout ratio of 0.5. The output softmax layer computes the probabilities of the input image being either a parasite or background.

Once the parasite candidates are extracted, we use a CNN model to classify them either as true parasites or background. In this work, we customize a CNN model consisting of seven convolutional layers, three max-pooling layers, three fully connected layers, and a softmax layer as shown in Fig. 6. A batch normalization layer is used after every convolution layer to allow a higher learning rate and to be less sensitive to the initialization parameters [29], followed by a rectified linear unit (ReLU) as the activation function [19]. Max-pooling layers are introduced after every two successive convolutional layers to select feature subsets. The last convolutional feature map is connected to three fully connected layers with 512, 50, and 2 hidden units, respectively. Between the three fully connected layers, two dropout layers [30] with a dropout ratio of 0.5 are applied to reduce model overfitting. The network is derived from VGG19 [27] by selecting the first six convolutional layers and three corresponding max-pooling layers from the VGG19 architecture to stop the feature maps at 64@5 × 5, followed directly by the fully connected and dropout layers. This shorter network structure provides similar performance while being faster and requiring less memory, which is important for smartphone applications. The output of the CNN model is a score vector, which gives the probabilities of the input image patch being either a parasite or background. We can obtain a larger or smaller number of predicted parasites by applying an adaptive probability threshold to the score vector.

Compared with pre-trained networks such as VGG [27], GoogLeNet [28], ResNet-50 [26], our customized CNN model has several advantages: 1) runtime is reduced by using a smaller set of customizable parameters, with the input size of the model being determined by the average parasite size in thick smear images (44 × 44 × 3), which is much smaller than the input size used by the other networks (224 × 224 × 3); 2) our smaller network structure with fewer layers is more suitable for smartphones. Since the input size is smaller, our network should in fact be less deep to avoid feature maps that are too small. A smaller network structure with less parameters also avoids over-training on the smaller input space. Compared to the pre-trained networks mentioned above, our customized CNN model achieves a better accuracy, despite having less network layers, and a shorter runtime. For an input image of 4032 × 3024 × 3 pixels, our system can complete the parasite detection within ten seconds (about eight seconds for candidate screening and two seconds for classification) on a standard Android smartphone. Both the smaller set of parameters and the smaller network structure contribute to the reduced runtime.

### C. Smartphone-Based Application

Based on the IGMS method and customized CNN model for parasite detection, we develop a smartphone-supported automated system to diagnose malaria in thick blood smears. We implement this system as a smartphone app for the Android mobile operating system. When using this app, the camera of the smartphone is attached to the eyepiece of the microscope. The user adjusts the microscope to find the target field in the blood smear and takes pictures with the app. The app then detects and counts parasites, records parasite numbers in a patient database, and displays the results in the user interface. Users will take several images until they have collected enough data to meet the requirements of their local protocols. The app will aggregate the parasite counts across all images taken. We implemented all algorithms using the OpenCV4Android SDK library.

**Fig. 7.**
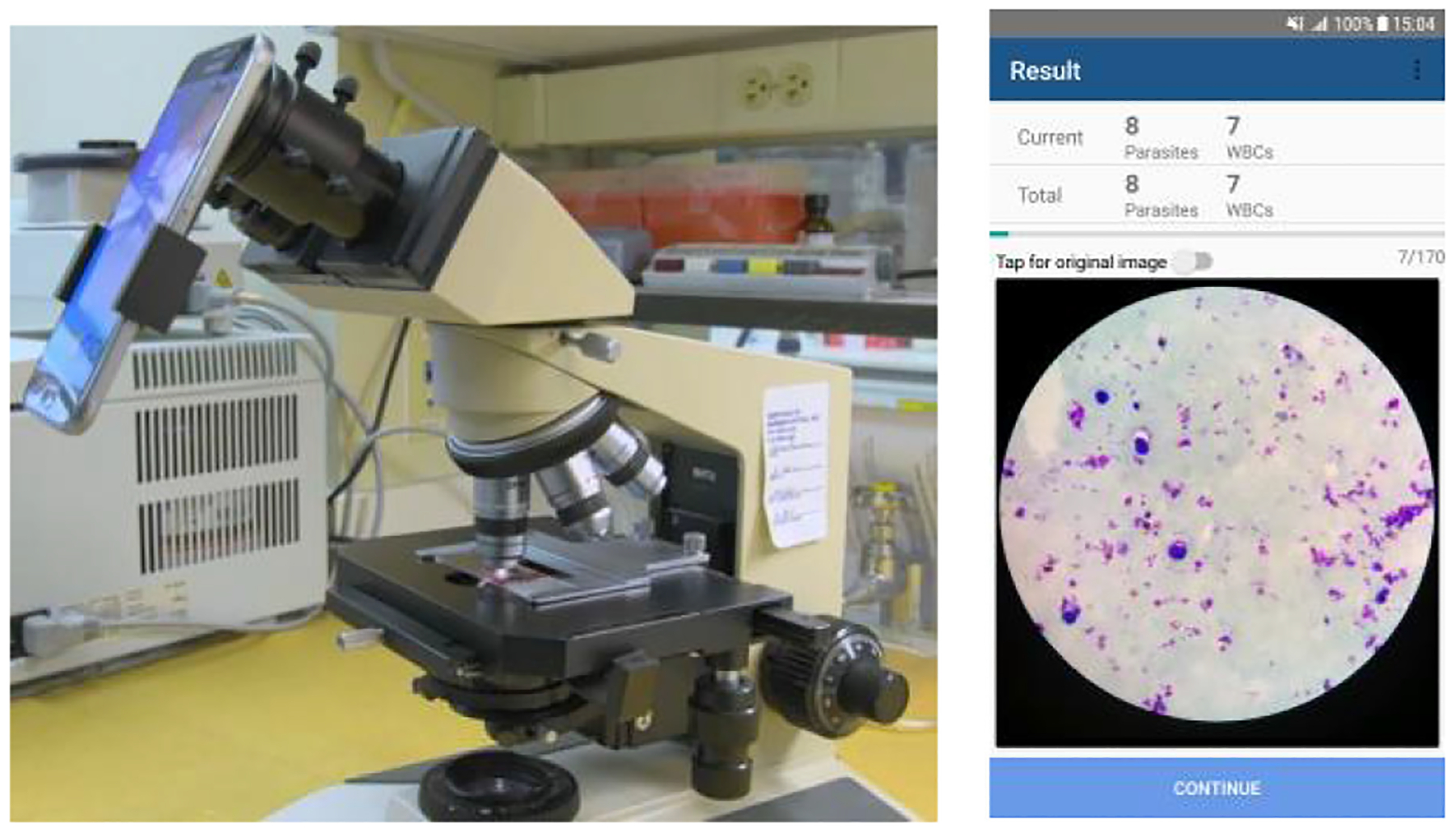
Smartphone-based malaria data acquisition and parasite detection.

After the image acquisition and processing stage, the app will go through a series of input masks for the user to fill in the information associated with the current patient and smear. This information is saved in the local database of the app, which we build with the SQLite API provided by Android. The app offers a user interface to the database where the user can view the data and images of previous smears, allowing hospital staff to monitor the condition of patients. Fig. 7 shows a smartphone running our app connected to a microscope (left-hand side) and a sample screenshot displaying a thick smear image with parasite counts (right-hand side).

## III. Data Preparation and Experimental Results

### A. Dataset

We photographed Giemsa-stained thick blood smear slides from 150 *P. falciparum* infected patients at Chittagong Medical College Hospital, Bangladesh, using a smartphone camera for the different microscopic field of views. Fig. 7 shows the smartphone-microscope setup and a screenshot of the phone displaying a thick smear image. Images are captured with 100x magnification in RGB color space with a 3024 × 4032 pixel resolution. An expert slide reader manually annotated each image at the Mahidol-Oxford Tropical Medicine Research Unit (MORU), Bangkok, Thailand. We de-identified all images and their annotations, and archived them at the National Library of Medicine (IRB#12972). In this work, we use 1819 thick blood smear images from these 150 patients. We publish the data here: ftp://lhcftp.nlm.nih.gov/Open-Access-Datasets/Malaria/Thick_Smears_150.

### B. Statistics of the Dataset

**Fig. 8.**
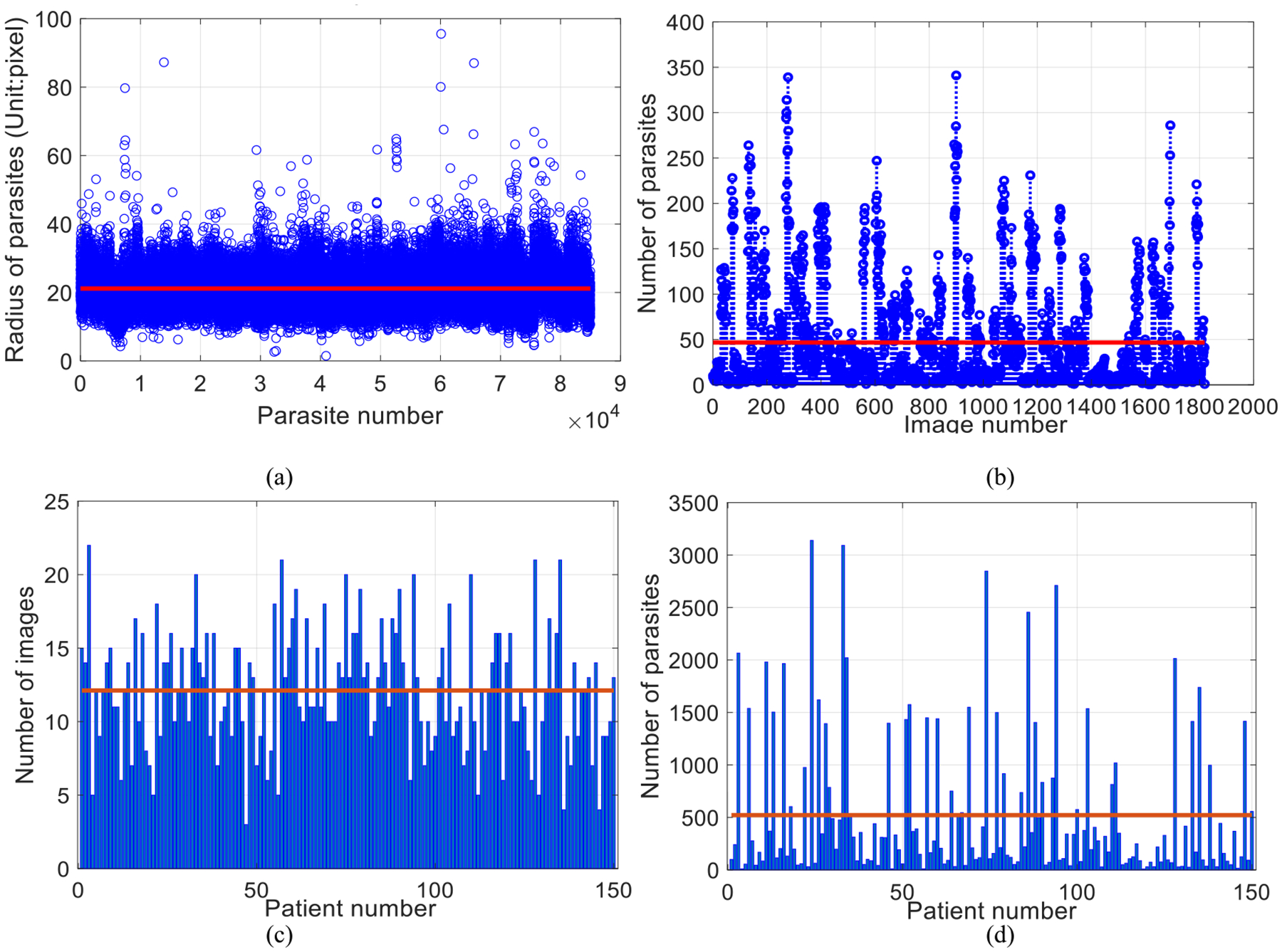
Statistical analysis of our dataset (including both Set A and Set B). The distribution of radii size of 84,961 parasites is plotted in (a), and the distribution of the number of parasites in 1819 images is illustrated in (b). The number of images and parasites in each patient is illustrated in (c) and (d) respectively. The red lines in the four subfigures indicate the average parasite radius, the average number of parasites in each image, the average number of images for each patient, and the average number of parasites for each patient, respectively.

We first perform a statistical analysis on the whole dataset of 150 patients. There are in total 84,961 annotated parasites, whose radius varies from two to 96 pixels, with an average radius of 22 pixels (Fig. 8(a)). Each image includes one to 341 parasites, with an average number of 47 parasites (Fig. 8(b)). Each patient set contains three to 22 images with an average number of 12 images (Fig. 8(c)), and contains eight to 3,130 parasites with an average number of 522 parasites (Fig. 8(d)).

### C. Data Partitioning

**Fig. 9.**
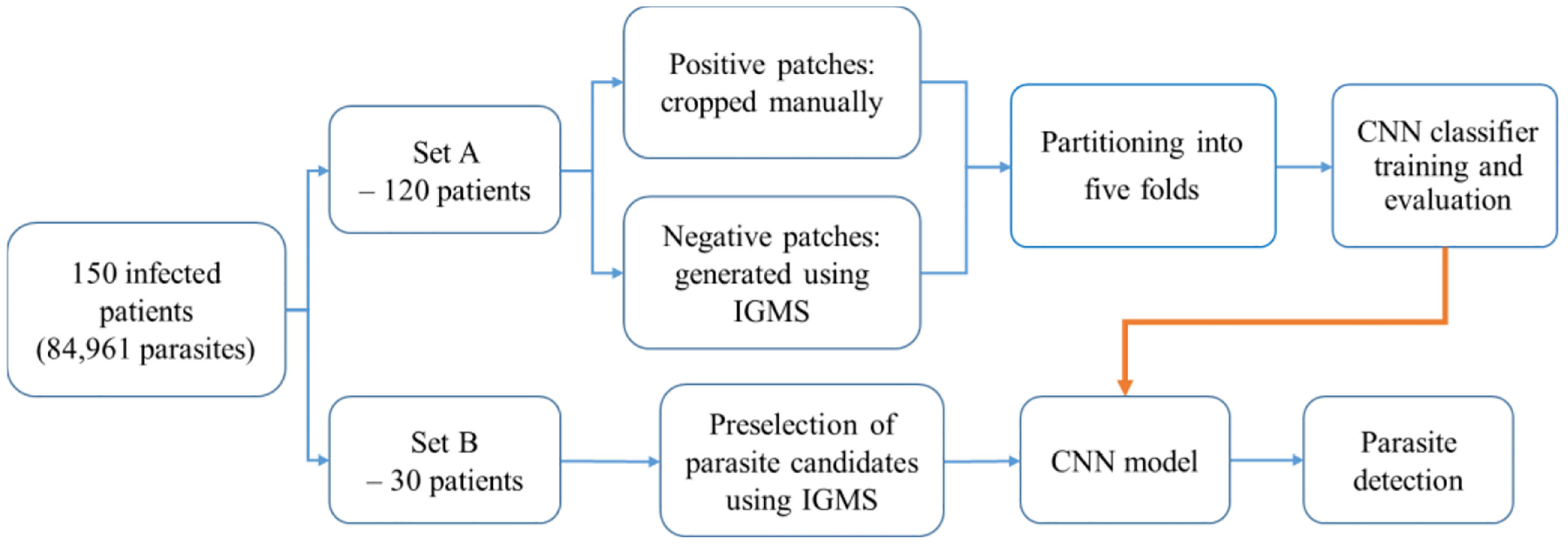
Data division strategy used in the experiments.

We divide the data on patient level into two sets: Set A and Set B, by a ratio of 4:1. Our data division strategy is shown in Fig. 9. Set A includes 120 patients with 1443 images and 72,184 parasites, and is used for the CNN model training and evaluation. Set B includes 30 patients including 375 images and 12,777 parasites, and is used for the performance evaluation of our method for automated parasite detection using screening and classification. We further split Set A into training sets and test sets on patient level, and evaluate the CNN model performance based on five-fold cross evaluation. To achieve a better performance for the CNN model, we use a balanced training set with an equal number of positive and negative patches. For each image in Set B, we generate 500 patches using IGMS.

### D. Preselection Performance

**Fig. 10.**
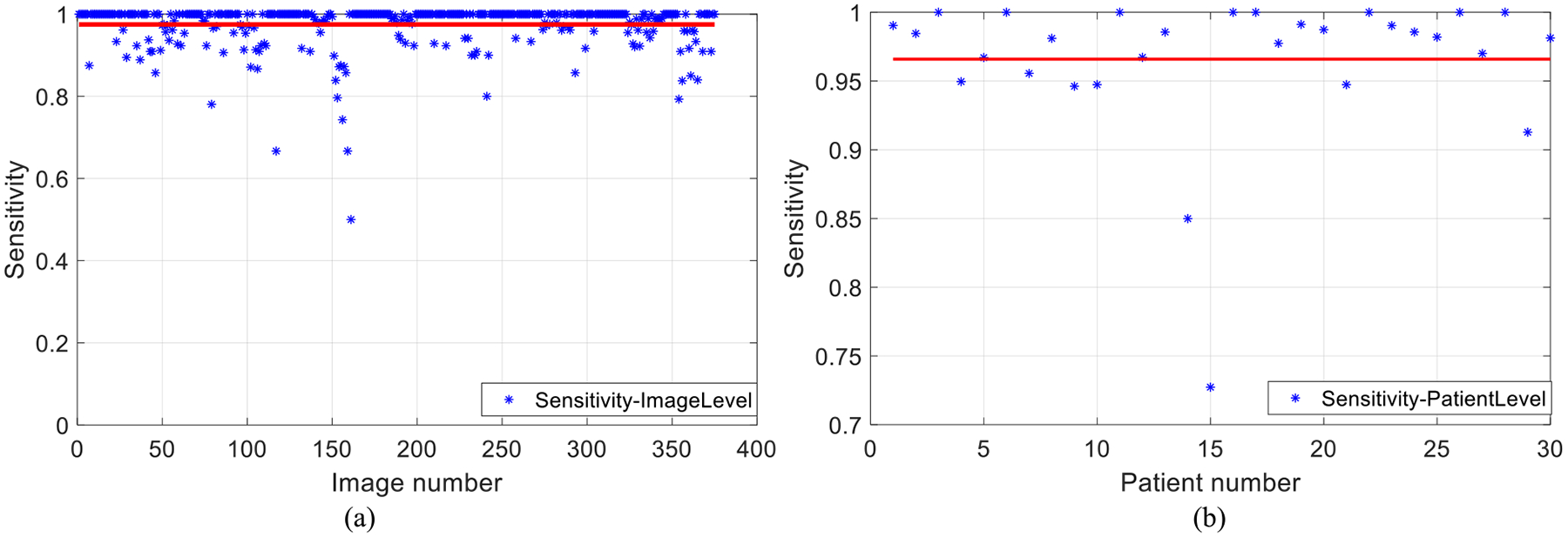
IGMS sensitivity of parasite preselection for 120 patients in Set B on image level (a) and patient level (b).

We evaluate the performance of IGMS as follows: We consider a parasite candidate generated by IGMS as a truly identified parasite if the overlap between it and the corresponding manually annotated parasite is larger than 50%. This overlap ratio is chosen empirically based on the balance of preselection sensitivity and classification accuracy. Then, we compute the sensitivity of IGMS as the ratio of the number of truly identified parasites to the total number of annotated parasites. Fig. 10 presents the sensitivity of IGMS on both image level and patient level for Set B. For parasite preselection, the proposed IGMS method achieves a sensitivity of 97.04% on patch level, 97.49% ± 5.40% on image level (Fig. 10(a)), and 96.59% ± 5.52% on patient level (Fig. 10(b)), respectively.

### E. Performance of the Customized CNN model

**Fig. 11.**
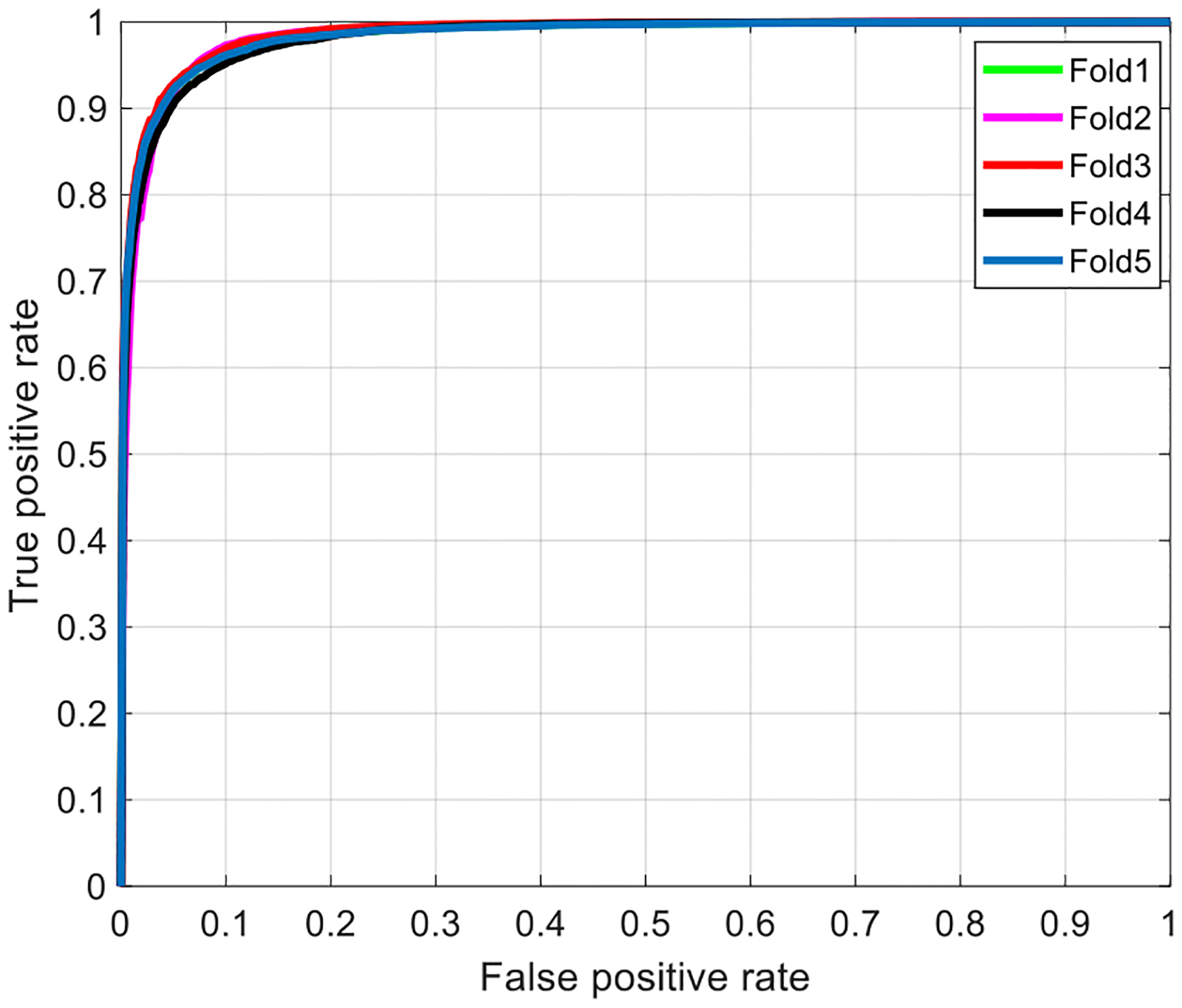
ROC curves of the customized CNN model with five-fold cross evaluation for Set A on patch level (AUC = 98.39% ± 0.18%).

We evaluate the performance of the customized CNN model on Set A using five-fold cross evaluation. Each fold contains 24 patients. Table II and Fig. 11 present the classification performance and receiver operating characteristic (ROC). According to Fig. 11, our customized CNN model achieves an average AUC score of 98.39%, and a standard deviation of 0.18%, showing its robustness and effectiveness. The average accuracy, F-score, specificity, sensitivity, precision, and negative predictive values for our customized CNN model are 93.46%, 93.40%, 94.33%, 92.59%, 94.25%, and 92.74%, respectively.

### F. Evaluation on Patch, Image and Patient Level

**Fig. 12.**
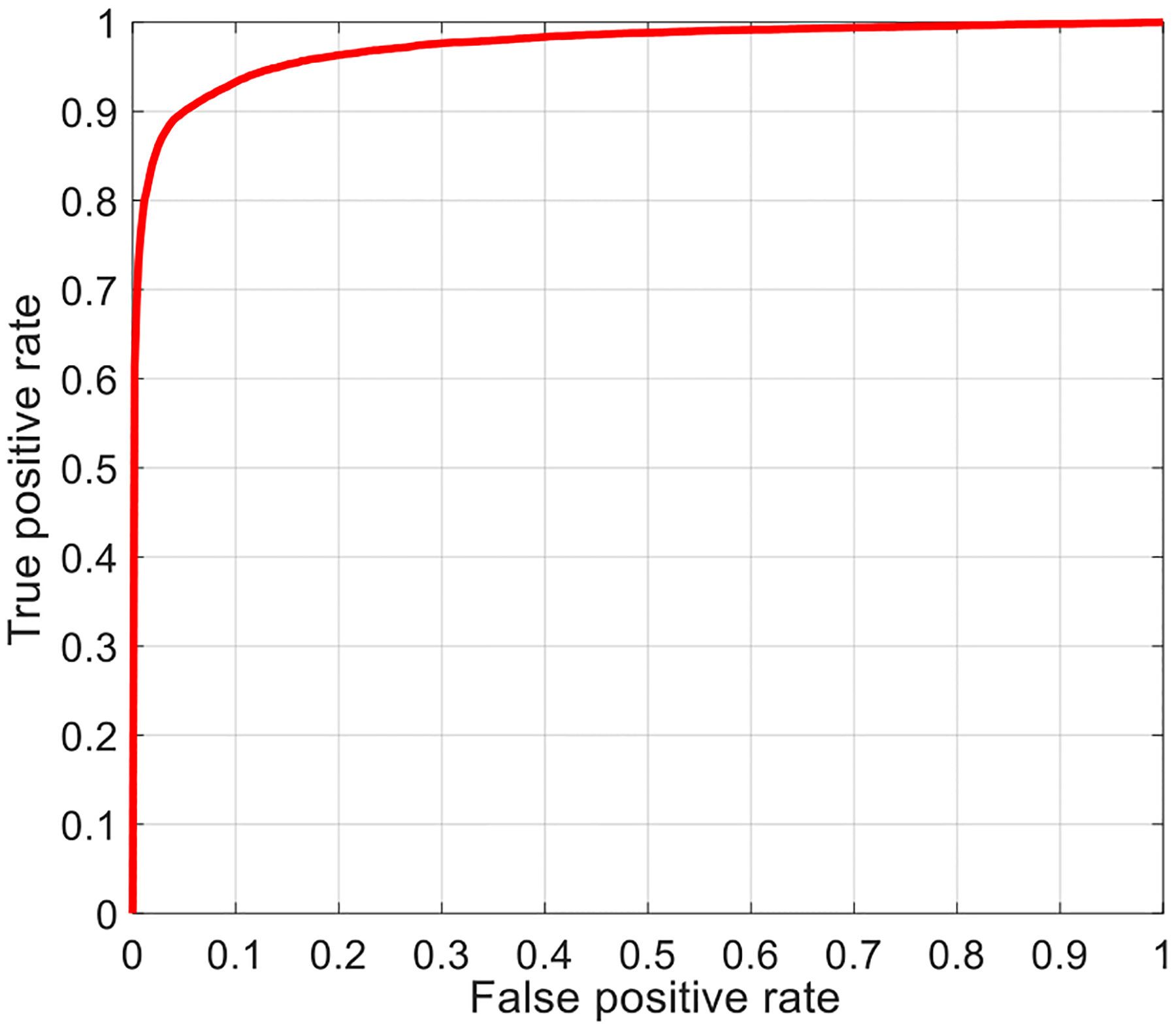
ROC curve of the proposed method on automatic parasite detection for Set B on patch level (AUC = 97.34%).

For the evaluation of our automated parasite detection method, we apply IGMS and CNN classifier to the 30 patients in Set B including 375 images and 12,777 parasites. Using IGMS, we generate 187,500 patches, among which 13,066 patches are considered positive because they overlap more than 50% with the ground truth annotations. Applying the customized CNN model to the 187,500 patches, we predict 13,687 patches as parasites, using a threshold of 0.6 for the classifier score. This threshold achieved the highest accuracy during the five-fold cross validation on Set A according to the ROC curve. For this threshold, we obtain the following performance metrics on patch level: accuracy 97.26%, AUC 97.34%, sensitivity 82.73%, specificity 98.39%, precision 78.98%, and F-score 80.81%. The corresponding ROC curve and confusion matrix are illustrated in Fig. 12 and Table III. From the ROC curve in Fig. 12, we see that we can achieve a sensitivity of 93% for a specificity of 90% by reducing the threshold of the classifier score.

**Fig. 13.**
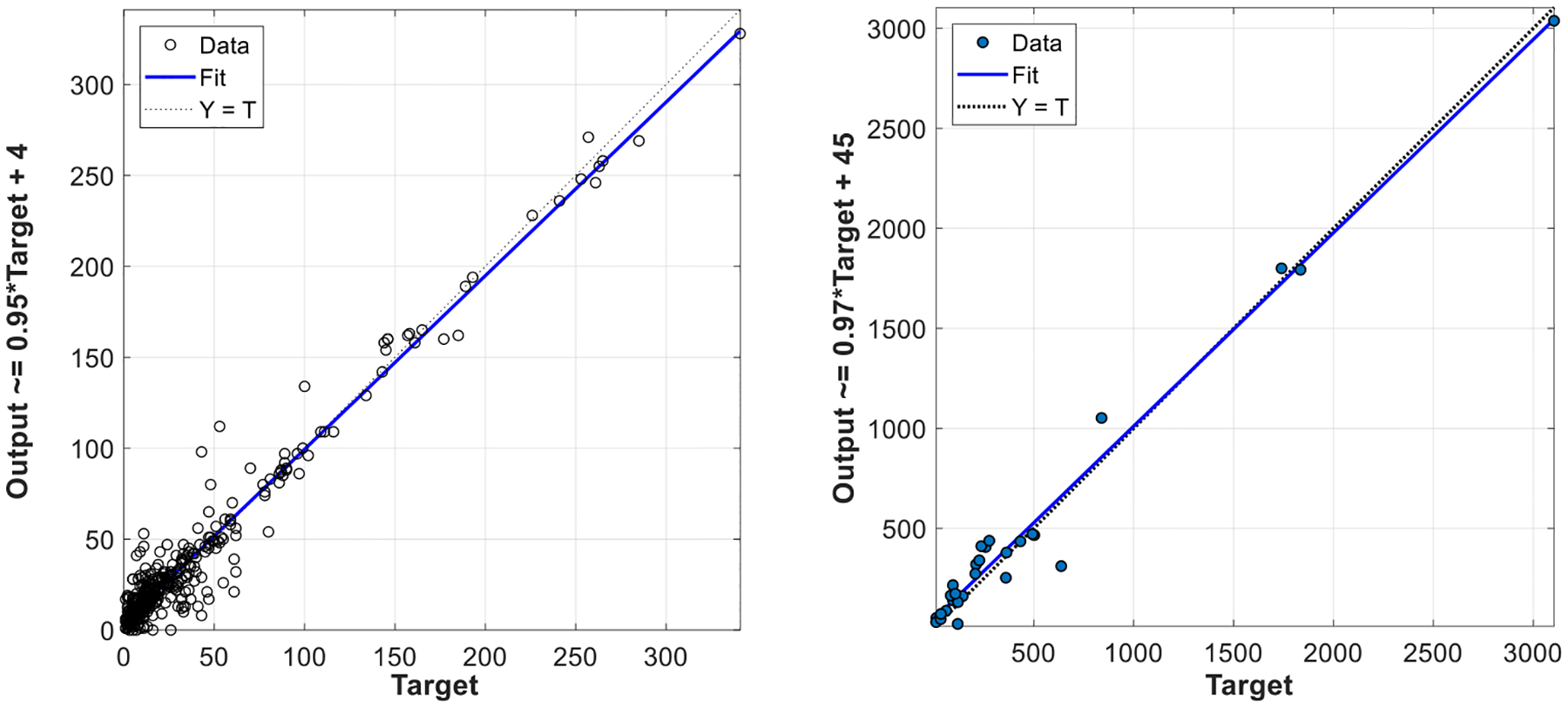
Linear regression between the number of automatically detected parasites (y-axis) and the number of parasites in the ground truth (x-axis) on image level (R = 0.98) (a) and patient level (R = 0.99) (b).

We also evaluate our method on both image level and patient level using linear regression, as shown in Fig. 13. On image level, we predict an average of 35 parasites in each image, with each image in the ground truth containing 34 parasites on average. A high correlation coefficient of R = 0.98 demonstrates the strong correlation between the number of predicted parasites and the ground truth Fig. 13(a). On patient level, we predict an average of 456 parasites for each patient, with an average of 426 parasites for each patient in the ground truth. We obtain a correlation coefficient of R = 0.99, which demonstrates a strong correlation on patient level as well, Fig. 13(b).

For an RGB image of 4032 × 3024 × 3 pixels, parasite detection takes about 10 seconds, including screening and classification, when implemented with TensorFlow Mobile on a Samsung Galaxy S6 (Exynos 7 Octa 7420 Processor, Android version 7.0).

**Fig. 14.**
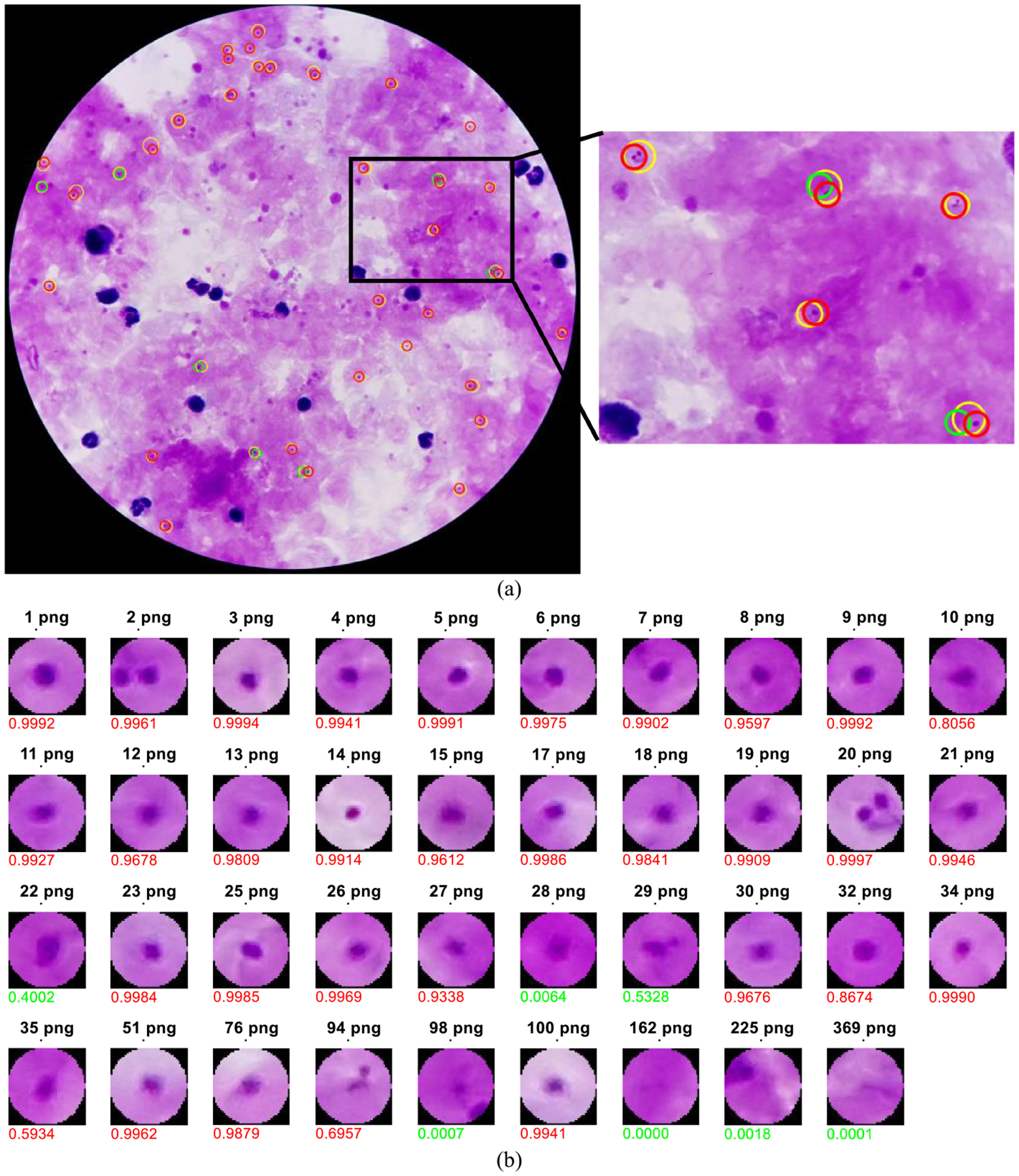
Parasite detection on an example image using our proposed method. (a) Parasites annotated in the ground truth (yellow circles) and screened parasite candidates that overlap more than 50% with the parasites in the ground truth (red and green circles). Red circles indicate candidates that are finally predicted as parasites (true preselected parasites), and green circles indicates those that are predicted as non-parasites (false preselected parasites). (b) Probabilities of parasite candidates that overlap more than 50% with parasites in the ground truth. The number under each patch denotes the output probability of the CNN. Red and green numbers indicate probabilities larger than 0.6 and smaller than 0.6, respectively.

Fig. 14 shows a practical example of parasite detection using the proposed method. In Fig. 14(a), parasites annotated in the ground truth are marked by yellow circles, whereas red and green circles indicate the screened parasite candidates (using IGMS) that overlap more than 50% with the parasites in the ground truth. Red circles indicate the screened candidates that are finally predicted as parasites (positives) by the customized CNN model, and green circles denotes those that are predicted as non-parasites (negatives) by the CNN model. In the enlarged rectangular region in Fig. 14, we observe five annotated parasites (yellow circles), and seven parasite candidates (red and green circles). Five of the parasite candidates are predicted as parasites by the customized CNN model. In Fig. 14(b), we list the probabilities for each candidate as given by the CNN.

### G. Comparison With Pre-Trained Networks

We perform evaluations on Set B to compare the performance between our method and pre-trained networks, such as AlexNet, VGG19, and ResNet50. First, we extract patch candidates using IGMS, and then apply different models to detect the true parasites. We compare performances in terms of accuracy, sensitivity, specificity, precision, F-score, AUC, and sensitivity for a given specificity. As listed in Table IV, the accuracy of our customized CNN model is about 1% higher than AlexNet, and about 4% higher than VGG19 and ResNet50. The F-score of our customized CNN model is about 5%, 15%, and 16% higher than the F-score of AlexNet, VGG19, and ResNet50, respectively. For a specificity of 98.39%, the sensitivity of our customized CNN is about 5%, 15%, and 23% higher than the sensitivity of AlexNet, VGG19, and ResNet50, respectively.

## IV. Discussion

In this work, we develop a smartphone-supported parasite detection application based on our IGMS method and deep learning. In Section III, we show that our application achieves an accuracy of 97.26% and an AUC of 97.34% on patch level, while obtaining a correlation coefficient above 98% on both image level and patient level. This is mainly due to two factors: First, the preselection of parasite candidates by IGMS covers parasites in the ground truth quite well. Second, our CNN model with the customized input size and network layers can classify the preselected candidates with high accuracy.

Our IGMS method generates false positive patches (negative patches) that are very similar to parasites (positive patches) so that our CNN model learns to reduce false positives. We have also performed experiments with negative patches randomly selected from the background. However, the accuracy decreased to less than 75% on Set B. This is because the random selection of negative patches generates too many clean negative patches. Therefore, training the CNN model on such patches leads to many false positives.

We have compared the performance of our method using three different input patch sizes: 36 × 36 × 3, 44 × 44 × 3 and 52 × 52 × 3. We observe that with a small patch size of 36 × 36 × 3, too many false positives are detected. Using this patch size, the method does not work because the patch size is too small to include enough information for identifying parasites. When using an increased patch size of 52 × 52 × 3, the AUC value on patch level is 97.30%, which is very close to our reported results for a patch size of 44 × 44 × 3. However, the correlation coefficients decrease to 0.96 and 0.97 on the image and patient levels. This is because more background noise is introduced when the patch size increases.

A comparison between the customized CNN model and a traditional SVM classifier based on HOG features shows that our customized CNN model outperforms the SVM classifier by about 6% in accuracy, 8% in sensitivity, 4% in specificity, 5% in precision, 6% in negative prediction, and 6% in F-score, respectively.

Based on the comparison between our customized CNN model and the three pre-trained networks Alexnet, VGG19, and ResNet50 (on a CPU), in Tables IV and V, we find: 1) our customized CNN model is more than ten times faster than VGG19 and ResNet50 (see Table V); 2) the accuracy of our customized CNN network is significantly better on Set A, between one and two percent, than the accuracy of a pre-trained VGG19 (p < 0.001) and AlexNet (p < 0.01), with a larger difference on Set B (Table IV). ResNet50 achieves an accuracy around 92.50% on Set A. However, ResNet50 is too big and too slow for our smartphone application; 3) according to the ROC curve, our customized CNN outperforms AlexNet, VGG19, and ResNet50 from 5% to 23% in terms of sensitivity for the given specificity. We have also applied object detection networks, such as faster-RCNN [31] and YOLO [32], to detect parasite candidates. However, these object detection networks do not work well for very small objects like parasites, with an average size of 44×44 pixels in an image of 4032 × 3024 pixels, resulting in many false negatives.

## V. Conclusion

In this paper, we implement a deep learning application for smartphones to detect malaria parasites in thick smear images. Our processing pipeline for automated parasite detection consists of two stages: parasite screening and classification. An intensity-based Iterative Global Minimum Screening (IGMS) first performs a fast screening of an entire thick smear image to generate parasite candidates. A customized CNN model then classifies each candidate as either parasite or background. Our experimental results demonstrate the practicality of our method for automatic detection of malaria parasites. To the best of our knowledge, our paper is the second paper that has developed a smartphone application for thick blood smear screening [18], and the first paper that has applied deep learning techniques for parasite detection in thick smears on smartphones, with evaluation on patient level. We make our dataset of 1819 images from 150 patients publicly available, as a service to the research community, which will mitigate the problem of lacking training data for automated malaria diagnosis in thick blood smears. Our future work is to improve the performance of our automated parasite detection method using network ensemble techniques and to improve its runtime on smartphones.

## Figures and Tables

**TABLE I:** Existing Approaches Applied to Parasite Detection in Thick Blood Smears

Authors	Methods	Images	Patients	SE/SP(%)	Remarks
Kaewkamnerd et al., 2011	Adaptive threshold on the V-value histogram + size filtering	20	-	60% DR	
Hanif et al., 2011	Intensity-based contrast enhancement + threshold-based segmentation	255	-	Qualitative results	Threshold is empirical
Chakrabortya et al., 2015	Color information based morphological segmentation	75	-	95% DR + 10% FPR	Patch level evaluation
Dave et al., 2017	Histogram-based adaptive thresholding + morphological operations on denoised images	87	-	91% DR	Patch level evaluation
Elter et al., 2011	Feature extraction from pre-detected plasmodia candidates + SVM classifier	256	-	97% SE	Patch level evaluation
Purnama et al., 2013	Feature extraction from RGB histogram + Genetic Programming classification	-	-	96% ACC	Only 180 patch images were used.
Yunda et al., 2011	Feature extraction from color, co-occurrence matrix related texture and wavelet-based texture + PCA feature reduction + neural network classification	110	-	76% DR	Patch level evaluation
Quinn et al., 2014	Feature extraction from connected components and moment features + randomized tree classifier	2903	133	20% SE + 90% PR	Patch level evaluation
Rosado et al., 2016	Adaptive thresholding + feature extraction in color and texture + RBF kernel based SVM classifier	94	6	80.5% SE +93.8% SP	Patch level evaluation
Delahunt et al., 2015	Parasite candidate localization + segmentation + feature extraction with CNN + linear SVM classification	-	143	LoD = 300 ~3000 p/μL + 92% SP	Patient level evaluation
Quinn et al., 2016	Small block splitting + CNN classifier	1182	-	97% PR	Patch level evaluation
Mehanian et al., 2017	Dynamic local thresholding for parasite candidate selection + SVM classifier + CNN classifier	1452	195	92% SE + 94% SP LoD =100 p/μL	Patch level and patient level evaluation
Torres et al., 2018	Same method as Mehanian et al., 2017		700	72% SE + 85% SP; 52% SE + 70% SP	Patient level evaluation

Note: DR indicates detection rate; FPR represents false positive rate; PR denotes precision; ACC means accuracy; SE indicates sensitivity; SP is specificity.

**TABLE II:** Classification Performance on Five Folds for Set A

Accuracy	F-score	Specificity	Sensitivity	Precision	Neg-pred
93.46%	93.40%	94.33%	92.59%	94.25%	92.74%
±0.32%	±0.33%	±1.25%	±1.27%	±1.13%	±1.09%

Note: Neg_pred is the negative predictive value.

**TABLE III:** Confusion Matrix for Set B on Patch Level

	Predicted Positive	Predicted Negative
Parasites	5.8%	1.2%
Non-parasites	1.5%	91.5%

**TABLE IV:** Performance of Different Networks on Set B

Network	Accu	Sensi	Speci	Preci	F-score	AUC	Sensil
IGMS+ResNet50	93.88%	81.34%	94.82%	54.04%	64.94%	95.48%	59.97%
IGMS+VGG19	93.72%	87.31%	94.20%	52.99%	65.95%	96.99%	67.26%
IGMS+AlexNet	96.33%	82.15%	97.39%	70.23%	75.73%	96.97%	77.77%
IGMS+Our CNN	97.26%	82.73%	98.39%	78.98%	80.81%	97.34%	82.73%

Note: Accu, Sensi, Speci, and Preci indicate accuracy, sensitivity, specificity and precision, respectively. Sensi1 is the sensitivity for a given specificity of 98.39%.

**TABLE V:** Performance Comparison Between the Customized CNN Model and DL Models in the Literature on Set A

Network	CPU or GPU	Learning rate	Training time	Accuracy
ResNet50	CPU	0.001	47382	92.55%
VGG19	GPU	0.001	13698	91.72%
AlexNet	GPU	0.001	1613	92.21%
Our CNN	GPU	0.001	1487	93.46%

## References

[1] “World malaria report,” WHO, Geneva, Switzerland, 2018.

[2] WHO, Guidelines for the Treatment of Malaria, 3rd ed. Geneva, Switzerland: World Health Organization, 2015.

[3] Makhija KS, Maloney S, and Norton R, “The utility of serial blood film testing for the diagnosis of malaria,” Pathology, vol. 47, no. 1, pp. 68–70, 2015. 25485654 10.1097/PAT.0000000000000190

[4] WHO, Malaria Microscopy Quality Assurance Manual. Geneva, Switzerland: World Health Organization, 2016.

[5] Poostchi M, Silamut K, Maude RJ, Jaeger S, and Thoma G, “Image analysis and machine learning for detecting malaria,” Transl. Res, vol. 194, pp. 36–55, Apr. 2018. 29360430 10.1016/j.trsl.2017.12.004 PMC5840030

[6] Liang Z , “CNN-based image analysis for malaria diagnosis,” in Proc. IEEE Int. Conf. Bioinf. Biomed, Shenzhen, China, 2017, pp. 493–496.

[7] Rajaraman S , “Understanding the learned behavior of customized convolutional neural networks toward malaria parasite detection in thin blood smear images,” J. Med. Imag, vol. 5, no. 3, Jul. 2018, Art. no. 034501. 10.1117/1.JMI.5.3.034501 PMC6050500 30035153

[8] Rosado L, Correia da Costa JM, Elias D, and Cardoso JS, “A review of automatic malaria parasites detection and segmentation in microscopic images,” Anti-Infective Agents, vol. 14, no. 1, pp. 11–22, Mar. 2016.

[9] Pattanaik PA and Swarnkar T, “Comparative analysis of morphological techniques for malaria detection,” Int. J. Healthcare Inf. Syst. Inform, vol. 13, no. 4, pp. 49–65, Oct. 2018.

[10] Kaewkamnerd S, Intarapanich A, Pannarat M, Chaotheing S, Uthaipibull C, and Tongsima S, “Detection and classification device for malaria parasites in thick-blood films,” in Proc. IEEE Int. Conf. Intell. Data Acquisition Adv. Comput. Syst, Prague, Czech Republic, 2011, pp. 435–438.

[11] Hanif NSMM, Mashor MY, and Mohamed Z, “Image enhancement and segmentation using dark stretching technique for Plasmodium Falciparum for thick blood smear,” in Proc. Int. Colloq. Signal Process Its Appl, Penang, Malaysia, 2011, pp. 257–260.

[12] Chakrabortya K, “A combined algorithm for malaria detection from thick smear blood slides,” J. Health Med. Inform, vol. 6, no. 1, pp. 179–186, Jan. 2015.

[13] Dave IR and Upla KP, “Computer aided diagnosis of malaria disease for thin and thick blood smear microscopic images,” in Proc. Int. Conf. Signal Process. Integr. Netw, Noida, India, 2017, pp. 4–8.

[14] Elter M, Hasslmeyer E, and Zerfass T, “Detection of malaria parasites in thick blood films,” in Proc. IEEE Eng. Med. Biol. Soc, Boston, MA, USA, 2011, pp. 5140–5144. 10.1109/IEMBS.2011.6091273 22255496

[15] Purnama IKE, Rahmanti FZ, and Purnomo MH, “Malaria parasite identification on thick blood film using genetic programming,” in Proc. Int. Conf. Instrum., Commun., Inf. Technol., Biomed. Eng, Bandung, Indonesia, 2013, pp. 194–198.

[16] Yunda L, “Automated image analysis method for p-vivax malaria parasite detection in thick film blood images,” Rev. S&T, vol 10, no. 20, pp. 9–25, Mar. 2011.

[17] Quinn JA, Andama A, Munabi I, and Kiwanuka FN, “Automated blood smear analysis for mobile malaria diagnosis,” in Mobile Point-of-Care Monitors and Diagnostic Device Design, Karlen W and Iniewski K, Eds., Boca Raton, FL, USA: CRC Press, 2014, pp. 1–20.

[18] Rosado L, Da Costa JMC, Elias D, and Cardoso JS, “Automated detection of malaria parasites on thick blood smears via mobile devices,” Procedia Comput. Sci, vol. 90, pp. 138–144, Dec. 2016.

[19] Krizhevsky A, Sutskever I, and Hinton GE, “ImageNet classification with deep convolutional neural networks,” in Proc. Advances Neural Inf. Process. Syst, Dec. 2012, pp. 1–9.

[20] Sun Y, Wang X, and Tang X, “Deep learning face representation from predicting 10,000 classes,” in Proc. IEEE Conf. Comput. Vision Pattern Recognit, Columbus, OH, USA, 2014, pp. 1891–1898.

[21] Ren S , “Rich feature hierarchies for accurate object detection and semantic segmentation,” in Proc. IEEE Conf. Comput. Vision Pattern Recognit, Boston, MA, USA, 2015, vol. 794, pp. 1–15.

[22] Delahunt CB , “Automated microscopy and machine learning for expert-level malaria field diagnosis,” in Proc. IEEE Global Humanitarian Technol. Conf, Seattle, WA, USA, 2015, pp. 393–399.

[23] Quinn JA, Nakasi R, Mugagga PKB, Byanyima P, Lubega W, and Andama A, “Deep convolutional neural networks for microscopy-based point of care diagnostics,” in Proc. Mach. Learn. Healthcare Conf, Los Angeles, CA, USA, 2016, pp. 271–281.

[24] Mehanian C, Jaiswal M, Delahunt C, and Thompson C, “Computer-automated malaria diagnosis and quantitation using convolutional neural networks,” in Proc. IEEE Int. Conf. Comput. Vision Workshops, Venice, Italy, 2017, pp. 116–125.

[25] Torres K , “Automated microscopy for routine malaria diagnosis: A field comparison on Giemsa-stained blood films in Peru,” Malaria J, vol. 17, no. 1, pp. 339–350, Sep. 2018. 10.1186/s12936-018-2493-0 PMC6157053 30253764

[26] He K, Zhang X, Ren S, and Sun J, “Deep residual learning for image recognition,” in Proc. IEEE Conf. Comput. Vision Pattern Recognit, Las Vegas, NA, USA, 2016, pp. 770–778.

[27] Simonyan K and Zisserman A, “Very deep convolutional networks for large-scale image recognition,” presented at the 3rd Int. Conf. Learn. Representations, San Diego, May 7–9, 2015, *arXiv1409.1556*.

[28] Szegedy C , “Going deeper with convolutions,” in Proc. IEEE Conf. Comput. Vision Pattern Recognit, Boston, MA, USA, 2015, pp. 1–9.

[29] Ioffe S and Szegedy C, “Batch normalization: Accelerating deep network training by reducing internal covariate shift,” in Proc. Int. Conf. Mach. Learn, Lille, France, 2015, pp. 81–87.

[30] Srivastava N, Hinton G, Krizhevsky A, Sutskever I, and Salakhutdinov R, “Dropout: A simple way to prevent neural networks from overfitting,” J. Mach. Learn. Res, vol. 15, pp. 1929–1958, Jun. 2014.

[31] Ren S, He K, Girshick R, and Sun J, “Faster R-CNN: Towards real-time object detection with region proposal networks,” IEEE Trans. Pattern Anal. Mach. Intell, vol. 39, no. 6, pp. 1137–1149, Jun. 2017. 27295650 10.1109/TPAMI.2016.2577031

[32] Redmon J, Divvala S, Girshick R, and Farhadi A, “You only look once: Unified, real-time object detection,” in Proc. IEEE Conf. Comput. Vision Pattern Recognit, Las Vegas, NV, USA, 2016, pp. 779–788.

[33] Otsu N, “A threshold selection method from gray-level histograms,” IEEE Trans. Syst., Man., Cybern, vol. SMC-9, no. 1, pp. 62–66, Jan. 1979.

